# R3DCID: circular interaction diagrams for RNA and DNA 3D structures of all sizes

**DOI:** 10.1261/rna.081031.126

**Published:** 2026-08

**Authors:** Patrick Nyadjo Fonga, Syed Muhammed Abubaker, Joey Thaman, Craig L. Zirbel

**Affiliations:** 1 Department of Mathematics and Statistics, Bowling Green State University, Bowling Green, Ohio 43403, USA; 2 St. John's Jesuit, Toledo, Ohio 43615, USA

**Keywords:** base stacking, base pair, DNA 3D structure, RNA 3D structure, secondary structure

## Abstract

There are over 21,000 atomic-resolution 3D structures containing RNA and/or DNA chains in the Protein Data Bank, ranging in size from 2 nt to tens of thousands. Most structures have more than one nucleic acid chain, and many have multiple models, assemblies, or symmetry operators. Nucleic acid 3D structures are formed by repetition of Watson–Crick base pairs and base stacking, but RNA (and increasingly DNA) is also shaped by non-Watson–Crick base pairs and various base-backbone interactions. It is difficult to comprehend the entirety of the interactions that form within and between chains in complex 3D structures; for example, there are several chains, over 5500 nt, and over 9700 pairwise interactions in one human ribosomal RNA 3D structure. Here we introduce R3DCID (RNA + DNA 3D structure Circular Interaction Diagram, pronounced “red sid”), a web service that produces circular arc representations of the pairwise interactions in all chains, models, assemblies, and symmetries in each nucleic acid structure in PDB. Diagrams have a uniform appearance and interpretation across all sizes and varieties of structures. Output is provided in PDF and SVG format for compact file sizes and smooth zooming and panning. R3DCID provides a new perspective on nucleic acid 3D structures; it serves as a guide to their overall structure and as a detailed map of the interactions that are critical to their function.

## INTRODUCTION

There are over 12,700 3D structures with DNA chains, over 9300 3D structures with RNA chains, and ∼300 with DNA/RNA hybrid chains deposited in the Protein Data Bank (PDB) (Burley et al. 2017). (Some structures have both RNA and DNA chains, so there is some double counting.) The structures range from 2 nt chains in complex with proteins to two-chain duplexes making Watson–Crick base pairs to tRNAs, ribozymes, viral capsids, viral RNA genomes, DNA origami structures, entire ribosomes, and multiple entire ribosomes with over 125,000 nt in a single structure file. Structures solved by NMR often have multiple models, as do a few structures solved by electron microscopy (EM). Some structures have coordinates for multiple biological assemblies, while others require symmetry operators to be applied to produce the entire 3D structure.

Visualizations are key to understanding 3D structures of biomolecules, and each visualization contributes a useful perspective. The PDB website provides high-quality visualizations of entire 3D structures, with the capability to show every atom and every atom-atom contact. Even so, 3D visualization is not the right tool for every purpose. For instance, large nucleic acid structures are hard to understand in their totality, and interactions within and between chains are difficult to track. Some key interactions are difficult to spot in a 3D structure, like non-Watson–Crick base pairs adjacent to a Watson–Crick double helix. Visualizations which produce a two-dimensional rendering to identify common interaction types, which we discuss below, are complementary to the 3D view.

Nucleic acids form a variety of pairwise interactions that have proven valuable to describe their internal structure, stability, and sequence variability. Watson–Crick base pairs are the most common, with their well-understood covariation being due to the GC and AT (or AU) base pairs being nearly perfectly isosteric (Stombaugh et al. 2009). There are also non-Watson–Crick base pairs, base-phosphate, and base-ribose interactions that are base specific and so contribute important constraints on sequence variability (Leontis et al. 2002; Zirbel et al. 2009, 2015). Base-base stacking and base-backbone oxygen stacking are also key recurrent features (St-Onge et al. 2007; Zirbel and Auffinger 2022).

An airport diagram (also called radial, or secondary structure diagram) is a common two-dimensional view of the Watson–Crick base pairs in a nucleic acid chain. An example is the well-known cloverleaf depiction of a tRNA, with the four-way junction in the center and the four helices radiating out from the junction, like the overhead view of an airport with four terminals extending from a central hub. In an airport diagram, the nucleotides are set along a curve which turns back on itself to bring into close proximity the nucleotides making Watson–Crick base pairs, often indicating GC with double lines, AU with a single line, and GU with a line and a closed dot in the center of the line, so that Watson–Crick double helices look like ladders. The set of Watson–Crick base pairs depicted this way is called the secondary structure, and the base pairs are said to be short-range or nested. Airport diagrams are well established for RNA and have the advantage that they can be drawn based only on a predicted set of Watson–Crick base pairs. In many cases, the original layout of the airport diagram for a molecule becomes a community standard, which facilitates comparison of homologous RNAs from different organisms. Some packages make it possible to produce airport diagrams with specific layouts, among them Exornata, XRNA, TRAVeler, R2DT, R2R, RiboDraw, and RNAViz (De Rijk et al. 2003; Weinberg and Breaker 2011; Elias and Hoksza 2017; Das and Watkins 2021; McCann et al. 2025; Meade et al. 2026). Other programs generate airport diagrams automatically, arranging helices coming out of junctions to avoid overlapping, including VARNA, Forna, RiboSketch, RNApuzzler, RNAcanvas, and RNAplot (Darty et al. 2009; Lorenz et al. 2011; Kerpedjiev et al. 2015; Lu et al. 2018; Wiegreffe et al. 2019; Johnson and Simon 2023). RNAview, RNAscape, and RNAproDB also generate diagrams where helices resemble ladders, arranged so their relative orientation matches the 3D structure as much as possible (Yang et al. 2003; Mitra et al. 2024, 2025b). Note that for larger structures these diagrams can be hard to follow. RNAview visualizations are available for small structures on the NAKB website (Lawson et al. 2024). DNAproDB displays DNA double helices in ladder format and shows details of protein contacts (Mitra et al. 2025a).

Airport diagrams can be used to display the interactions found in a 3D structure, but they have important limitations and drawbacks. First, in RNA especially, there are often “long-range” interactions between distant parts of the diagram. For example, in tRNA the common G19-C56 base pair (numbering may differ in other 3D structures) occurs between the hairpin ends of the D-arm and T-arm, which extend in opposite directions from the central junction in an airport diagram. When multiple such Watson–Crick base pairs occur in succession the interaction is called a pseudoknot. Long-range interactions and pseudoknots can be added to an airport diagram automatically, but then they tend to run over other parts of the diagram, or they can be added and routed manually. Second, many programs only display Watson–Crick base pairs, even though non-Watson–Crick base pairs can account for ∼1/3 of the base pairs in RNA molecules. Those which enable the display of non-Watson–Crick base pairs, among them Exornata, R2DT, VARNA, R2R, RNAcanvas, RiboSketch, RNAscape, and RNAproDB, still face the challenge of long-range base pairs cluttering up an airport diagram when added automatically, and which take a long time to add manually. Third, no program that we are aware of adds base stacking or base-backbone interactions to airport diagrams, even though these interactions are plentiful and important. Fourth, airport diagrams are rarely used to show multiple chains in the same complex, with the exception of the 5.8S and 5S ribosomal RNAs and some cases with self-cleaving ribozymes. Overall, airport diagrams put most of the focus on nested Watson–Crick base pairs and they minimize the display of non-Watson–Crick base pairs and other interactions, even though those are critical to the formation and stability of the full 3D structure.

Arc diagrams provide additional 2D visualizations of base pairs. A linear arc diagram arranges all nucleotides along a horizontal line and indicates base pairs with circular arcs above the line. Helices appear as nested sets of arcs, and pseudoknots cross over the nested arcs. When two secondary structures are under consideration (either because of uncertainty in prediction or because of dynamic changes between secondary structures), showing one set above the line and the other below makes it easy to compare secondary structures, as done by R-CHIE, RNAbows, and RNAvigate (Lai et al. 2012; Aalberts and Jannen 2013; Irving and Weeks 2024). Circular arc diagrams are similar, but with the nucleotides arranged around a circle and the base pair arcs inside the circle. Circular arc diagrams are used to compare two or more secondary structures by CS^2^BP^2^-Plot and RNAStructViz (Léger et al. 2019; Schmidt et al. 2021). DNAproDB makes circular arc diagrams with protein contacts shown (Mitra et al. 2025a). Some programs including VARNA, RNAStructViz, RNAvigate, produce linear and circular arc diagrams in addition to airport diagrams. Note that linear and circular arc diagrams are not designed to put nucleotides in close proximity when they are close in physical space, or to reflect the mutual orientations of nucleotides on the diagram.

In this paper, we introduce a new web-based tool called R3DCID, pronounced /red sid/, which creates circular arc diagrams showing pairwise interactions for all RNA and DNA 3D structures from PDB. R3DCID can display on a single diagram all nucleotides in all models, assemblies, chains, and symmetry operated nucleic acid chains found in the structure file, plus solitary nucleotides bound to protein chains. R3DCID provides a uniform presentation of the interactions in all PDB files, including structures with over 125,000 nt and cases with 60 symmetry operators. With default parameters, the R3DCID diagram shows all base pairs, base stacking, and base-backbone interactions as circular arcs. Output formats are PDF and SVG, which are vector graphics formats with native circular arc commands, which keeps file sizes small and allows for highly performant zooming and panning to see fine details. The web interface makes it easy to modify the display parameters and output format.

R3DCID diagrams complement the atomic 3D view by annotating pairwise interactions between nucleotides, including those between chains in multichain complexes such as ribosomes. They serve as a guide to the contents and interactions in each structure, and enable comparison between models and assemblies in the same molecule, and between instances of the same molecule from different experiments or from different organisms. As with airport diagrams, helices stand out clearly in R3DCID diagrams, but pseudoknots and the many other long-range interactions are easier to follow in R3DCID, and multichain structures are visualized easily. Moreover, ribosomes and other selected structured RNAs have helices automatically numbered by R3DCID following standard numbering from airport diagram templates, to facilitate reference to airport diagrams and comparison between species.

## RESULTS

**FIGURE 1.**
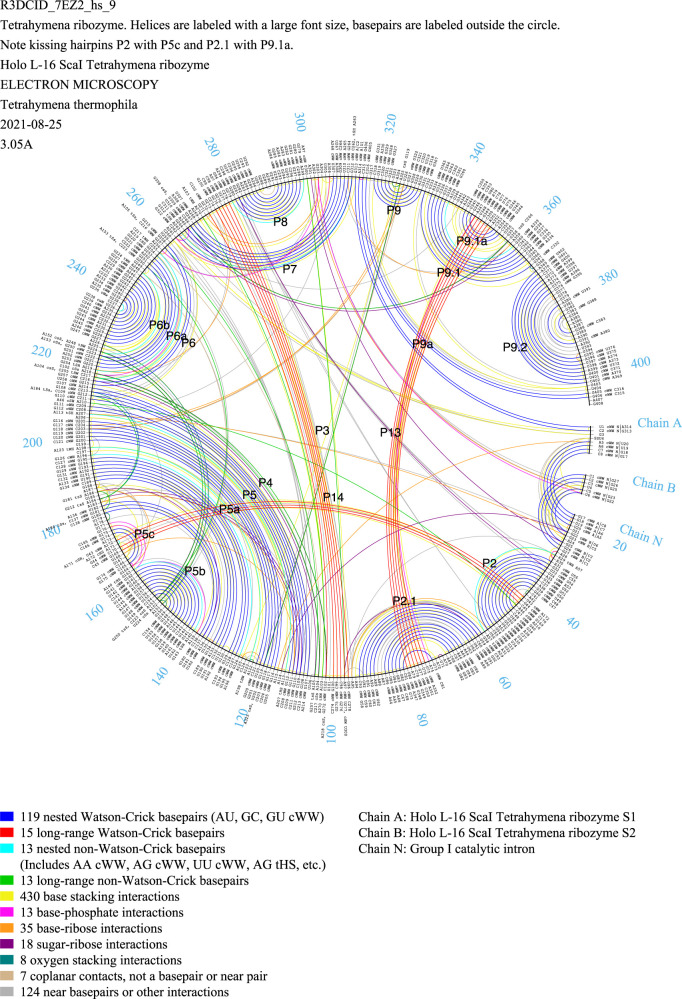
Circular interaction diagram for the tetrahymena ribozyme structure 7EZ2 (Su et al. 2021). The diagram uses default coloring, shows all arcs, helix numbers with font size 9, and displays a listing of base pair interactions around the outside of the circle. The legend below the diagram indicates how often each type of interaction occurs and the color of the corresponding arcs. The legend also lists descriptions of the three chains. This diagram is Example 1 on the R3DCID input page https://rna.bgsu.edu/fr3d/r3dcid and also appears on page 1 of the Supplemental Material.

R3DCID is a web server that produces circular arc diagrams in PDF or SVG format, to represent the nucleotides in RNA or DNA 3D structures and the pairwise interactions that they make. First we describe the results using default settings for a diagram with one chain. More complex situations are described further below, and optional display parameters are described in the next section. See Figure 1 for reference.

Nucleotides are arranged clockwise around an outer black circle on the diagram. Circular arcs are drawn inside the outer circle between interacting nucleotides, with colors indicating the type of interaction. The arcs are layered based on interaction type, starting with near interactions in light gray, on top of that coplanar contacts in tan, then stacking interactions in yellow, followed by base-backbone interactions: backbone oxygen stacking interactions in dark green, sugar-ribose interactions in purple, base-ribose interactions in orange, base-phosphate interactions in magenta (Zirbel et al. 2009, 2015; Abu Almakarem et al. 2012; Zirbel and Auffinger 2022). Nested non-Watson–Crick base pairs are drawn next in cyan, and then long-range non-Watson–Crick base pairs; these include all Leontis–Westhof base pairs besides the canonical AU, GC, GU base pairs (Leontis et al. 2002). Then nested Watson–Crick AU, GC, and GU pairs in dark blue, the most common base pairs, and finally the long-range Watson–Crick base pairs, also known as pseudoknots, in red. In this way, the helices formed by Watson–Crick base pairs are prominent, with occasional pseudoknots highlighted on top of those.

Each nucleotide on the diagram is labeled outside the circle with the base and author-assigned nucleotide number, like A140. Base pair interactions made by the nucleotide are listed on the same line of text, indicating the interacting partner for clarity. Leontis–Westhof nomenclature is used, which indicates the interacting edges and the orientation of the glycosidic bonds, for example A140 tHS G163 indicates that nucleotide A140 makes a trans Hoogsteen-Sugar edge base pair with G163 in the same chain. The same interaction could also be listed as G163 tSH A140. The chain is labeled outside the circle at the start of the chain using the author-assigned chain name, up to four characters. For example, “Chain B.”

Above the diagram, the header lists the PDB identifier, the structure title, the experimental method, the release date, the biological source, and the reported resolution. Below the diagram and to the left, counts of each interaction type and a box indicating the arc color appear in a legend. Below the diagram and to the right is a list of each distinct chain in the structure and a text description of it.

### Chains, models, symmetries, assemblies

Most structures have more than one chain. The chains are indicated around the outside of the diagram by separate black circular arcs; see Figure 1. The chains are ordered alphabetically in the absence of additional considerations. When the chain sequences match an Rfam ribosomal family, chains are put in this generic order: 5S, 5.8S, LSU, SSU, mRNA, tRNA, clockwise around the circle (Ontiveros-Palacios et al. 2025). This order keeps the interaction arcs between chains as short and nonoverlapping as possible. See Supplemental Material pages 2, 3, 7, 8, 9 for diagrams for ribosomal structures. Supplemental Material page 9 shows the ribosomal large subunit from *Toxoplasma gondii* where ribosomal subunits are broken across 32 smaller chains, with the LSU chains ordered alphabetically. Comparison to other LSU diagrams shows the same overall structure. Alphabetical ordering is not used in a small number of cases where chains are better ordered next to their Watson–Crick pairing partners; see Supplemental Material page 12.

NMR and EM files can have dozens of models, and the interactions may vary from model to model. The models are arranged in numerical order around the outer circle, and within each model the chains are ordered as above. Model 1 chain B would be indicated by “Chain 1|B” outside the circle. See Supplemental Material page 5 for an example with seven models and page 14 for an example with 15 models.

Many 3D structures require symmetry operators to be applied to the coordinates of one or more chains in the PDB file to generate all coordinates in the structure. The default symmetry is 1_555, and others have identifiers such as 2_656; see Supplemental Material pages 6 and 11. Symmetry operated chains of this type are ordered first to keep Watson–Crick paired chains together, and after that in order of their symmetry operator. Some structures have symmetry operators that are simply numbered and appear in the format P_3 or ASM_5, for example, viral capsid structures often have 60 symmetry operators; see Supplemental Material page 13. Numbered symmetries are arranged in numerical order around the diagram, and within each symmetry operator, chains are ordered as above. Chains with symmetry operators other than 1_555 are indicated with the symmetry operator following the chain identifier, for example “Chain B 2_656.”

Many 3D structures have more than one biological assembly. By default, all assemblies are shown, in numerical order around the circle. Chains and symmetries within each assembly follow the order described above. Assembly 1 chain B is labeled “A1 Chain B.” Viral capsids often have assembly 1 with 60 symmetry operators, assembly 2 to show the asymmetric unit, assembly 3 with symmetry operators 1, 2, 3, 4, 5 to show the meeting of five symmetry-generated units, then assembly 4 with operators 1, 2, 6, 10, 23, 24 to show the meeting of six units. See Supplemental Material page 13 for an example of a viral capsid with four assemblies, many symmetry operators, and stem–loops which contact each other through all types of base-backbone interactions.

The legend below and to the right of the diagram lists each chain in the order in which it first appears in the diagram. Chains which are mapped to an Rfam family are listed with their “standardized” name. For example, chains A and B of PDB entry 5BTP has rcsb_polymer_entity, pdbx_description “RNA (62-MER),” which is not descriptive (Jones and Ferré-D'Amaré 2015). The chain sequences map to Rfam family RF01750, whose name is “ZMP-ZTP.” We display the standardized name “ZMP/ZTP riboswitch.” When no standardized name is available, the chain is listed with its pdbx_description value.

For ribosomes and a selected few other molecules, helix numbers are displayed at the apex of the arcs indicating Watson–Crick base pairs in each helix. For ribosomes, these helix numbers match the numbering in use since 1983 and as posted with the RiboVision suite (Maly and Brimacombe 1983; Bernier et al. 2014; McCann et al. 2024). Helix numbers are very helpful for navigating ribosomal structures across phylogenetic domains plus mitochondria and chloroplast ribosomes. See Materials and Methods for the numbering in reference ribosomal structures and the method of mapping from reference structures to other structures of the same molecule. For selected nonribosomal structures, we use the helix numbering from the publication; see Figure 1 for an example.

Some chains in some structures have nucleotides that were part of the experimental setup but are not resolved with *xyz* coordinates. Those nucleotides are shown inside parentheses, like (A1234), with the base followed by its numerical position in the experimental sequence, starting at 1, because there is no author-assigned nucleotide number for these nucleotides. Sometimes this numbering sequence is out of sync with the author-assigned numbers for nucleotides with *xyz* coordinates. Optionally, nucleotides without coordinates can be omitted; the omission is marked with a small break in the outer black circle. See Supplemental Material page 2 after U2098 in Helix 76 for the continuation of the chain sequence where coordinates are not available, and page 3 for a chain break after C3948 in Helix 76 due to nucleotides without coordinates being omitted.

Some structures have nucleotides that are associated with a protein chain, often in the role of a ligand bound to the protein. They are not designated as nucleic acid chains, but they still sometimes make base pairs with nucleotides in nucleic acid chains. R3DCID endeavors to identify such solitary nucleotides, display them, and indicate the interactions they make; see Supplemental Material page 10 for an example. When the solitary nucleotide is associated with a nucleic acid chain, it is shown at one end of the chain after a small break in the outer black circle; see Supplemental Material page 16 for an example.

**FIGURE 2.**
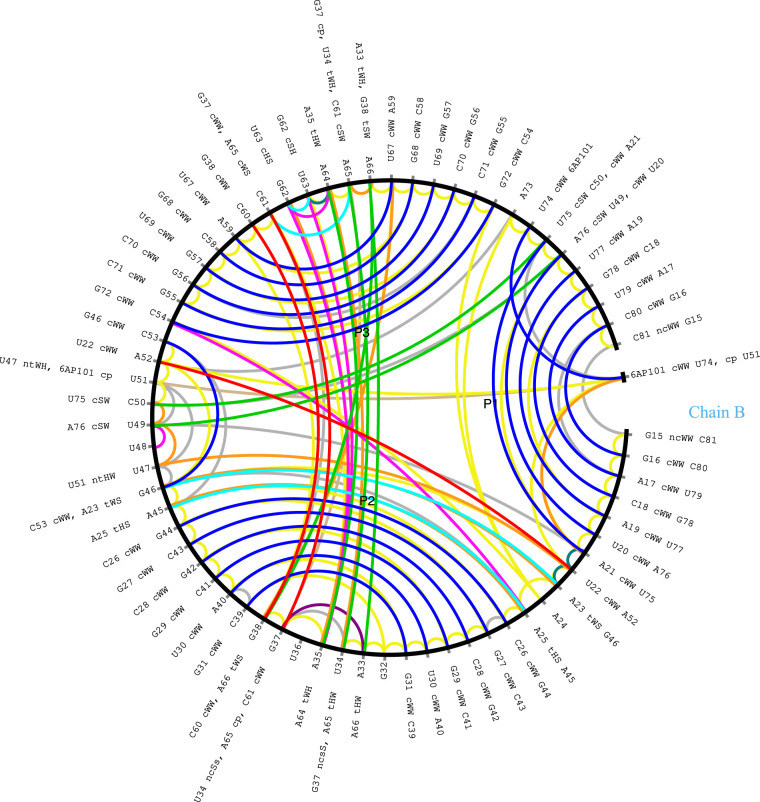
Circular interaction diagram for the purine riboswitch structure 4FEO chain B (Stoddard et al. 2013). The diagram uses default coloring, shows all arcs, helix numbers with font size 9, and displays a listing of base pairs, near base pairs, and coplanar contacts around the outside of the circle. Modified nucleotide 6AP101 is associated with chain B but not covalently bonded to it. This diagram is Example 16 on the R3DCID input page https://rna.bgsu.edu/fr3d/r3dcid and appears on page 16 of the Supplemental Material.

Modified nucleotides are indicated around the circle with their chemical component dictionary identifier, which can be from one to five characters. See modified nucleotide SSU in Chain A in Figure 1 and 6AP in Figure 2. The pairwise interaction annotator used by RNA 3D Hub covers over 800 modified nucleotides, as listed on the NAKB Modified Nucleotide page https://www.nakb.org/modifiednt.html. We annotate most cases of modified nucleotides making base pair, base stacking, and base-backbone interactions, but coverage is not yet 100%. Hybrid chains contain both RNA and DNA nucleotides, and these are handled with no difficulty; see Supplemental Material page 15 for an example.

R3DCID generates output in PDF and SVG formats. Both are vector graphic formats that permit infinite zooming without loss of resolution, and both have circular arcs as native commands, keeping file sizes small. Large files with many nucleotides can be displayed. The largest at the moment is 4V3P, with 125,580 nt from 23 complete eukaryotic ribosomes generated by 23 symmetry operators, making almost 230,000 pairwise interactions; there are a small number of contacts between the different ribosomes which are indicated on the diagram (Myasnikov et al. 2014). Second largest is 7AS5, a DNA nanostructure with 32,459 nt across 445 chains (Kube et al. 2020). Third is 6WLN, an EM structure with 80 models of the same chain of length 349; unusually, nucleotide numbering begins at -10 (Kappel et al. 2020). Fourth is 4V4G, with 22,350 nt from five ribosomes in five different assemblies (Vila-Sanjurjo et al. 2004).

### Example: Tetrahymena ribozyme

In Figure 1 we show the R3DCID output for the Tetrahymena ribozyme from PDB entry 7EZ2 (Su et al. 2021). We use default parameters, except for the helix label font size, which is set to 9 to be large enough for a publication figure. There are three chains, A, B, and N, which interact via Watson–Crick base pairs. The text around the outside of the circle indicates all nucleotides present in each chain and the base pairs they make. Every 20th nucleotide is indicated with a large cyan number around the outside of the circle. There are three pseudoknots within chain A shown with red arcs, two of which are kissing hairpins. Nested double helices, shown in blue, and the pseudoknots have helix labels as in the original publication. Note the long-range non-Watson–Crick base pairs, shown in green, between hairpin P5b and the end of helix P6a; this has the typical appearance of a long-range A-minor interaction. That interaction involves A153 making a cSS base pair with C223, which makes a Watson–Crick (cWW) base pair with G250, which in turn makes a tSS base pair with A153. Note that the 2 bp made by C223 are listed as “A153 cSs, G250 cWW C223” next to the tick mark for C223. Overall, stacking interactions are most numerous, followed by the nested Watson–Crick base pairs; all other interactions do not add up to the number of Watson–Crick interactions, but are critical to the molecule achieving the required 3D structure. The same structure is shown on page 1 of Supplemental Material.

### Example: Purine riboswitch

Purine riboswitches are RNA aptamer domains of ∼70 nt which occur in the 5′ UTRs of certain messenger RNAs in many bacteria. They regulate transcription and translation by binding adenine, guanine, or similar ligands. In Figure 2 we show R3DCID output for purine riboswitch chain B from PDB structure 4FEO (Stoddard et al. 2013). Main features of the structure are: three helices (labeled P1, P2, P3), two hairpin loops making a pseudoknot of two Watson–Crick base pairs (G37 with C61 and G38 with C60) and a number of long-range non-Watson–Crick base pairs, and a three-way junction. Nucleotides in the three single-stranded linkers between the helices form a number of interactions, including two isolated Watson–Crick pairs that cross one another (G46 with C53, marked as nested, and U22 with A52, marked as long-range), cSW pairs (U49 with A76 and C50 with U75), and a tWS pair (A23 with G46). The bound ligand is 6AP, a variant of adenine, which is a solitary residue listed in the PDB file as being a member of chain B even though it has no backbone atoms and is not covalently bonded to chain B. The outer black circle in Figure 2 indicates 6AP 101 as being part of chain B but not covalently bonded. The colored arcs show that it makes multiple contacts with the three single-stranded linker regions, including a Watson–Crick base pair with C74, coplanar interaction with U51, base-ribose interaction with A21, and stacking interactions with U22, A52, and U75. These interactions form the binding pocket for the purine-like ligand. Figure 2 illustrates the possibility to make a diagram with no header, no legend of interaction counts, and no chain listing. This diagram is Example 16 on the R3DCID input page and page 16 of Supplemental Material.

We show R3DCID output for 35 purine riboswitch chains, each with a distinct ligand, on pages 16–50 of Supplemental Material, to illustrate the use of R3DCID to compare multiple chains to one another. The 35 purine riboswitch structures were selected and sorted as follows. We started with the Representative Set of RNA 3D Structures release 4.37, dated April 29, 2026, at the 4.0A resolution threshold. We filtered on Rfam clan CL00123, which includes Rfam families RF00167 (Purine riboswitch aptamer) and RF01510 (2dG-I riboswitch aptamer), resulting in 100 chains whose sequences map to these families. Among these are structures of the Guanine-II riboswitch (Li et al. 2025). We omitted chains with length longer than 100 due to their different overall structure, leaving 91 chains of length around 70. We sorted these by Composite Quality Score CQS2, which accounts for resolution and other structure quality factors as explained at https://rna.bgsu.edu/rna3dhub/nrlist. We kept the first example with each distinct ligand. Some of the 31 ligands are most similar to A, others to G, and there are numbering differences between the structures. In Supplemental Material we display first the 10 structures with a ligand that maps to A, all of which pair with position numbered 74 (6AP, A2F, 3AW, ZZR, 3AY, ZZS, ADE, 29G, 2BP, N6M, pages 16–25), then the 10 with ligands that map to G and make a Watson–Crick pair with C74 (HPA, 5AZ, Q44, GUN, CMG, ANG, 6GU, 29H, DX4, 6GO, pages 26–35), then the nine that map to G and pair with C64 (XAN, GMP, GNG, NPR, OXG, 9QC, 7PD, H4B, A1LXN, pages 36–44), then two examples of the 2dG-I riboswitch, whose ligand pairing partner is numbered 80 (DGP, 5GP, pages 45–46), then four with no ligand bound (pages 47–50).

Comparison of the 35 purine riboswitch circular interaction diagrams shows conservation of the features mentioned above for 4FEO|1|B: three helices, two hairpins making a two-pair pseudoknot and other contacts, crossing isolated Watson–Crick pairs between linker strands (except for the Guanine-II riboswitch on Supplemental Material pages 37–44 and the structures with no ligand on pages 47 and 48), one or two cSW pairs, and a tWS pair as in 4FEO|1|B. The ligands nearly all have an edge that resembles a purine Watson–Crick edge and which pairs with the nucleotide numbered 74/64/80. The bound ligands all make a coplanar interaction or near base pair with the nucleotide corresponding to U51 in 4FEO|1|B. The coplanar annotation is especially helpful here because the ligands generally lack the ribose moiety, allowing interactions with their sugar edge that are not seen in nucleic acid chains, and so which have not been incorporated into the Leontis–Westhof annotation system. The ligands generally make the same stacking interactions as in 4FEO|1|B, but the base-ribose interaction with the analog of A21 is present in only 16 of the 31 structures with a bound ligand.

### Input page and parameter selection

The input page for R3DCID (available at https://rna.bgsu.edu/fr3d/r3dcid) provides a flexible interface for specifying the content of a Circular Interaction Diagram. The user can elect to view the SVG file directly in the browser or download a PDF output file. To facilitate viewing of R3DCID output online, we provide an HTML viewer for the SVG output. The HTML viewer provides for infinite zoom using key controls, mouse wheel, or touchpad, and one can click and drag to pan. We provide a link at the top of the HTML viewer to a web page that lists the base pairs and provides a coordinate visualization window to examine each pair, as posted on the RNA 3D Hub site. From there, one can easily see all other interaction types and their coordinates.

Entering a single PDB identifier (e.g., 1ABC) is sufficient to generate a diagram containing all associated chains with default parameters as described above. When more selective visualization is desired, users can specify particular models and chains using a structured syntax. For instance, 1ABC|1|A will display chain A from model 1. Multiple chains may be listed following the format 1ABC|1|A,B,C which displays chains A, B and C from model 1. Similarly, entering 1ABC|3,2,1|A will display chain A from models 3, 2, 1 in that order, and 1ABC|1,2,3|A,B will display both chains from all three models. When greater control is needed, R3DCID allows specification of models and chains together, for example 1ABC|1|A + 1ABC|2|B + 1ABC|3|C to see chain 1 from model 1, chain B from model 2, and chain C from model 3. These specifications follow the RNA 3D Hub convention for unit identifiers starting pdbid|model|chain; see https://www.bgsu.edu/research/rna/help/rna-3d-hub-help/unit-ids.html for more information.

Similarly, users may select one or more biological assemblies and include symmetry-generated molecules when appropriate. For example, entering 4,2 in the Assemblies field instructs R3DCID to display assemblies 4 and 2 in that order, as on page 7 of Supplemental Material. The same principle applies to the Symmetries field: entering multiple symmetries separated by commas includes the corresponding symmetry-generated molecules in the diagram. Detailed syntax and additional examples are provided on the Circular Interaction Diagram Help page.

Beyond structure selection, the interface provides these ways to customize the diagram:

- Select which header fields to show; default is the structure title, experimental method, biological source, release date, and resolution.
- Add a custom text description to the header.
- Choose among three color schemes, the default described above, a colorblind-safe palette (see Supplemental Material page 4), and grayscale (see Supplemental Material page 3).
- Select which categories of interaction arcs are shown, dimmed, or hidden, including arcs within or between chains.
- Decide which interaction types, and helix membership, are listed as text outside the circle.
- Display or suppress nucleotides whose atomic coordinates were not resolved in the experiment.
- Adjust the font size of helix numbers overlaid on the arcs; helix size 0 suppresses this display.
- Display or omit a legend with interaction counts and/or the listing of chains and their names.

The 16 examples on the R3DCID input page show various choices of the display parameters. They generate diagrams which are made available in PDF format in Supplemental Material pages 1–16. The examples range in size from a small DNA duplex to complete ribosomes.

### Availability

#### Web server

The R3DCID web server is free to use and has no registration requirement. It is available at https://rna.bgsu.edu/fr3d/r3dcid.

#### URL input and reproducibility

The input page encodes all selections directly into a URL, and the R3DCID server acts on the URL. Thus, each generated diagram is fully reproducible and shareable by using the URL. Direct URL input is also supported, for example, to generate and download diagrams programmatically, or to embed the SVG file on a web page. A comprehensive explanation of the URL input syntax and parameter options is provided on the R3DCID Help page, where each feature is described in greater detail with illustrative examples.

When a request is made from the web server, the SVG and PDF files are cached on the web server for faster retrieval in the future. However, files with user-added descriptions are generated new each time, never served from the cache, so the text of the description field is not available to any other user.

#### Python code

R3DCID is written in Python. The source code is posted in the fr3d-python GitHub repository https://github.com/BGSU-RNA/fr3d-python/ under the flask folder. It is covered by the MIT license. The program r3dcid.py can be run locally but requires internet access to download information about chains, assemblies, models, Rfam mappings, and pairwise interactions. Use the ‐‐help option to see the command line arguments.

### Limitations

R3DCID only produces diagrams for RNA and DNA-containing 3D structures posted on PDB, and it only shows pairwise interaction annotations generated by the RNA 3D Hub.

## DISCUSSION

We chose PDF and SVG because they are vector graphic formats, allowing high zooming without loss of visual quality. The PDF and SVG files maintain the visual integrity of our diagrams at all scales, ensuring all details are preserved accurately. All rendering choices are made in order to produce small output files that can be viewed, zoomed, and panned across with excellent performance. Circular arcs are used because they look good and they can be drawn with a single native PDF or SVG command. PDF files are compressed and stored in binary format, keeping file sizes small. A eukaryotic ribosome circular diagram in PDF format can be ∼800 kb; bacterial ∼600 kb. Uncompressed SVG file sizes are larger, but compressed SVG is typically smaller than PDF. In contrast, the Python matplotlib and cairo packages generate PDF files using Bezier curves, which result in a much larger file size and slower rendering.

In practice, SVG in a browser works well up to the size of a ribosome. PDF in a browser works to somewhat larger sizes but usually only allows zoom up to 500%. For ribosomes and larger structures, a dedicated PDF viewer works best, because it may allow zooming up to 6400% and zooms and pans better, even for very complicated diagrams with thousands of nucleotides and their interactions.

When the arcs for Watson–Crick base pairs cross one another, a determination must be made which ones to consider “nested” and which ones are “long-range.” Different methods for making this determination have been delineated and each has its advantages; we choose the IR (incremental, range) method (Smit et al. 2008) We start with the Watson–Crick base pairs between nucleotides that are closest to each other in the chain and mark those as nested, and when we encounter Watson–Crick base pairs that cross those, we mark them as long range. As a result, sometimes a short “nested” Watson–Crick pair is crossed by a pseudoknot making more base pairs, which may be more dynamically stable; at any rate these cases are visually clear, and the display facilitates reflection on the fact that either could form first in the folding pathway of the molecule.

It is common for a single nucleotide to make many different interactions simultaneously, 1 or 2 bp, two or three base-base stacking interactions, and possibly multiple base-backbone interactions. Arcs for different interaction types are slightly offset from one another so that multiple interactions made by the same nucleotide do not overlap completely and can always be seen. Note that most base pairs and near base pairs also make coplanar contacts; in R3DCID diagrams we only include coplanar contacts that are not annotated as base pairs or near base pairs. Coplanar contact annotations often reveal single hydrogen bond contacts in base triples or other contacts between base edges that can only form in a specific structural context.

R3DCID offers a powerful tool to study complex RNA molecules from the Protein Data Bank. Unlike existing tools, R3DCID can handle very large RNA structures and visualize multiple chains simultaneously, highlighting critical inter-chain interactions. The input page allows researchers to customize diagrams. The diagrams make it possible to conduct detailed analyses of RNA and DNA interactions and facilitate comparisons between structures, which complements examination of individual structures. By accurately depicting various 3D interactions in 2D circular diagrams, including base-pairing, base-stacking, and base-backbone interactions, R3DCID facilitates a deeper understanding of the details of RNA and DNA 3D structure.

## MATERIALS AND METHODS

The RNA 3D Hub pipeline at BGSU retrieves new RNA and DNA 3D structures from the PDB each week shortly after they are released. They are annotated with base pairs, base stacking, base-backbone interactions, and coplanar contacts using the program NA_pairwise_interactions.py in the fr3d-python repository. See https://rna.bgsu.edu/rna3dhub/pages/interactions for additional information about the annotation types. Information about models, assemblies, symmetries, and chains is extracted from each PDB file or downloaded from PDB. All data is stored in the RNA 3D Hub database. Note that assembly membership is from the first released version of the mmCIF file posted at PDB; in some cases like 5J7L the assembly numbers are different in later versions available from PDB.

For this project, we developed Python code r3dcid.py which accepts a PDB identifier and optional selection parameters such as specific chains, models, and/or assemblies in the 3D structure. When only a PDB identifier is provided, it uses an API from RNA 3D Hub to retrieve all chains in the structure which contain nucleic acids. It uses another API to get the sequence of nucleotides in the experimental structure; we refer to this as the experimental sequence, which may be longer than the list of observed nucleotides having *xyz* coordinates. The program downloads precomputed annotations of pairwise interactions between nucleotides. Using precomputed annotations makes R3DCID much faster.

The R3DCID web server is implemented using flask. The flask deployment files are available in the fr3d-python GitHub repository alongside r3dcid.py.

When PDF output is desired, r3dcid.py generates PostScript code, which is then converted into PDF using ps2pdf from the ghostscript package (Artifex Software, GhostScript. I. https://artifex.com/). The code also generates SVG commands in a text file, which is displayed directly by web browsers. When producing the PostScript and SVG commands, we take care to draw all arcs of the same color at the same time, reducing file size and rendering time for color changes. Similarly, all text of the same size is drawn at the same time. We draw arcs from back to front instead of using layers for simplicity. As the number of nucleotides on the diagram increases, we use a larger PDF page size so that one can zoom in to the nucleotide level; sufficient zoom may only be available with a dedicated PDF viewer. For example, a eukaryotic ribosome will typically have page width and height doubled; see pages 3 and 7 in Supplemental Material. A PDB file with over 100,000 nt uses a page size 10 times US letter size. The page size does not impact zooming with SVG files; the HTML viewer allows for unlimited zooming.

Rfam maps PDB chains to Rfam families in each Rfam release, with the results appearing in the Structures tab on each Rfam family page (Ontiveros-Palacios et al. 2025). During the weekly RNA 3D Hub pipeline run, the sequence of each chain in new PDB structures is scored against the covariance models for all Rfam families, as described in an earlier publication (Roll and Zirbel 2026). When an Rfam mapping is made, a “standardized name” is assigned to the chain, such as “Large subunit ribosomal RNA.” These names are more consistent than the names of Rfam families, which often also indicate the domain. Standardized names are also more consistent than the pdbx_description field.

Ribosomal helix numbers were recorded for chains in selected PDB files using ranges posted for the RiboVision2 project on the GitHub page https://github.com/LDWLab/Ribovision_2.0_GT/tree/master/populate_db/Associated_Data (McCann et al. 2024). We manually adapted the numbering to additional reference PDB files from different domains. The manual helix number assignments can be seen for each available Rfam family in the annotations directory of the fr3d-python GitHub repository. Assignments are transferred from reference structures to other structures using alignments of PDB chain sequences made with Rfam covariance models, as previously described (Appasamy and Zirbel 2024). R3DCID uses an API provided by RNA 3D Hub to retrieve the transferred helix numbers for each chain; the API follows the format https://rna.bgsu.edu/correspondence/nucleotide_annotation?chain=7K00|1|a for each available chain.

## SUPPLEMENTAL MATERIAL

Supplemental material is available for this article.

## Figures and Tables

**Patrick Nyadjo Fonga:**
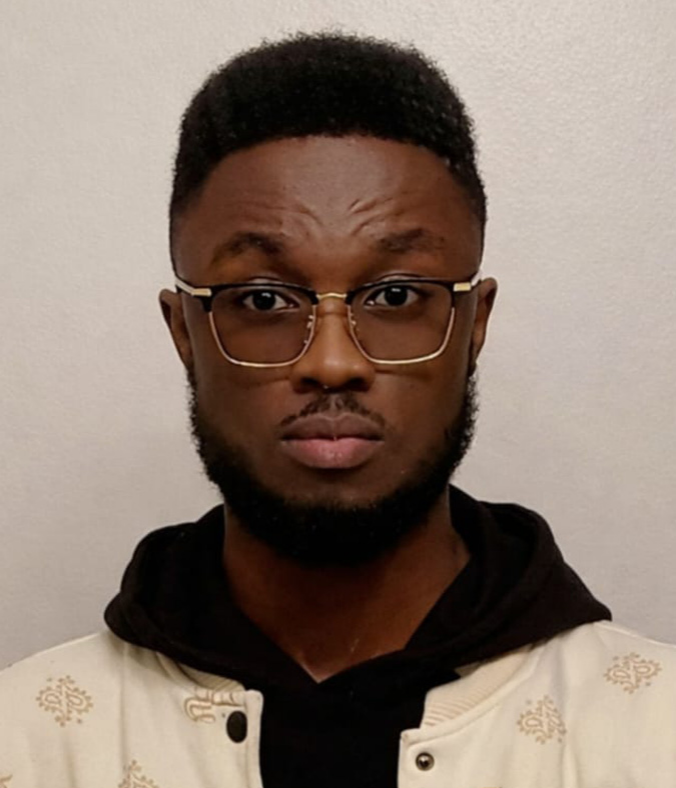


## MEET THE FIRST AUTHOR

***Meet the First Author(s)* is an editorial feature within *RNA*, in which the first author(s) of research-based papers in each issue have the opportunity to introduce themselves and their work to readers of *RNA* and the RNA research community. Patrick Nyadjo Fonga is first author of this paper, “R3DCID: circular interaction diagrams for RNA/DNA.” Patrick is a PhD candidate in mathematics at Bowling Green State University, specializing in functional analysis and operator theory. He is also a research assistant working under the supervision of Craig Zirbel, supported by the National Institute of General Medical Sciences of the National Institutes of Health, where he contributes to the analysis and visualization of RNA and DNA 3D structures.**

**What are the major results described in your paper and how do they impact this branch of the field?**

R3DCID introduces a web-based tool that generates circular interaction diagrams for RNA and DNA 3D structures. The major result is that R3DCID can represent, in a compact two-dimensional format, base pairs, stacking interactions, base–phosphate interactions, base–ribose interactions, and other nucleotide contacts across single chains, multiple chains, models, biological assemblies, and symmetry-generated molecules. Its main impact is that it enables the visualization of complex nucleic acid structures containing dozens to thousands of nucleotides and interactions in a clear, uniform, and scalable 2D format, complementing traditional 3D visualization approaches.

**What led you to study RNA or this aspect of RNA science?**

I came to study RNA through my involvement in a research project on RNA base triples. While working with these data sets, it became clear how challenging it is to understand all the interactions within large RNA and DNA structures using standard 3D visualizations. This led to an interest in developing tools that can provide a clearer, more global view of these interactions.

**During the course of these experiments, were there any surprising results or particular difficulties that altered your thinking and subsequent focus?**

One of the main challenges was our goal of making the diagrams as complete as possible. We quickly realized how many different types of interactions and structural features needed to be considered, and it became difficult to decide what to prioritize. A key difficulty was finding the right balance between completeness and readability, capturing as many meaningful interactions as possible while avoiding an overwhelming or cluttered representation. This led us to focus on designing a visualization that remains informative and interpretable, highlighting the most relevant interactions while preserving a coherent global structure.

**If you were able to give one piece of advice to your younger self, what would that be?**

I would advise my younger self to focus less on finding immediate answers and more on understanding the problem deeply. Clarity often comes gradually, especially in interdisciplinary work.

**What are your subsequent near- or long-term career plans?**

My long-term goal is to pursue an academic career in mathematics that integrates both research and teaching. I aim to contribute to the development of mathematical theory while also exploring meaningful connections with other disciplines, and to engage students in a rigorous study of mathematics.
